# Towards a Genetic Linkage Map of the California Condor, an Endangered New World Vulture Species

**DOI:** 10.3390/ani12233266

**Published:** 2022-11-24

**Authors:** Michael N. Romanov, Yang Da, Leona G. Chemnick, Steven M. Thomas, Sugandha S. Dandekar, Jeanette C. Papp, Oliver A. Ryder

**Affiliations:** 1 Conservation Genetics, Beckman Center for Conservation Research, San Diego Zoo Wildlife Alliance, Escondido, CA 92027, USA; 2 Department of Animal Science, University of Minnesota, Saint Paul, MN 55108, USA; 3 Human Genetics Department, GenoSeq Core, University of California, Los Angeles, CA 90095, USA

**Keywords:** California condor, linkage map, microsatellite loci, conservation genomics

## Abstract

**Simple Summary:**

The California condor is a critically endangered representative of New World vultures maintained under restoration and reintroduction programs. Within a California condor genome research project, we made a preliminary step toward a genetic linage map for this iconic bird species. The respective linkage data were generated using a panel of 121 condors. The condors were genotyped for 123 polymorphic microsatellite markers. The condor genotyping and mapping results are a useful addition to the previously obtained physical and cytogenetic maps and can be further utilized in condor genome sequence assembly.

**Abstract:**

The development of a linkage map is an important component for promoting genetic and genomic studies in California condors, an endangered New World vulture species. Using a set of designed anonymous microsatellite markers, we genotyped a reference condor population involving 121 individuals. After marker validation and genotype filtering, the genetic linkage analysis was performed using 123 microsatellite loci. This resulted in the identification of 15 linkage groups/subgroups that formed a first-generation condor genetic map, while no markers linked to a lethal chondrodystrophy mutation were found. A panel of polymorphic markers that is instrumental in molecular parentage diagnostics and other genetic studies in the California condor was selected. Further condor conservation genomics research will be focused on updating the linkage map and integrating it with cytogenetic and BAC-based physical maps and ultimately with the genome sequence assembly.

## 1. Introduction

Among New World vultures (Aves; Cathartidae), the California condor (*Gymnogyps californianus*) is a spectacular landmark species, being the largest bird in North America. It has, however, a critically endangered status [1] and is subject to a long-term restoration and reintroduction program (e.g., [2,3,4,5]).

The genetic management of the California condor population is one of the cornerstones of the whole conservation effort for this endangered avian species [6,7,8]. In the course of the restoration of the current condor population, a lethal chondrodystrophy condition was revealed that is inherited as a recessive autosomal character [6,9]. The condor conservation program will greatly benefit from the genetic mapping and characterization of this deleterious mutation [9,10]. For this purpose, we created a suite of genomic resources and tools including a California condor microsatellite-enriched library [9,10,11], a genomic BAC library and a BAC-based chicken-condor comparative physical map [9,12], and molecular cytogenetic maps [13,14]. There were few research reports that have employed a sizable quantity of microsatellite markers to examine a variety of genetic factors and phenomena, including parentage and the proof of facultative parthenogenesis in California condors [10,15,16]. The segregation of microsatellites (or sequence-tagged site markers) essentially characterizes a genome linkage map (e.g., [17]).

Recently, the California condor genome was sequenced [18,19,20] resulting in more prospects, promises, and instruments for the investigation of the genomic features and emerging genetic conditions of condors, while ensuring that the condor conservation will take advantage of genome studies [21]. As stated by Lewin et al. [22], “*every genome sequence needs a good map,*” which implies the development and integration of genetic linkage and cytogenetic and BAC-based physical maps, and the subsequent alignment of these elements with a genome sequence. As has been proven for other genome projects (e.g., [17,23,24]), the map integration and sequence alignments are necessary steps for verifying, correcting, and improving the initial genome assembly of a particular species and its individual chromosomes. This is also true for the first-generation genome assembly of the California condor that still lacks information on microchromosomes 10–11 and requires a further chromosome assignment of 512 unplaced/unlocalized scaffolds totaling a ~52.2 Mb sequence [25], as well as the verification of the assembled chromosome sequences for errors and the subsequent corrections. Therefore, further mapping efforts and elaboration will facilitate further progress in condor genomics.

Previously, we completed a preliminary genetic diversity assessment and linkage analysis in the condor population using 17 anonymous polymorphic microsatellite loci [10]. For that study, we selected a group of 121 related condor individuals that constituted a condor resource population. We found that the average number of alleles was 2.41 per locus, the average heterozygosity for all loci was 0.45, and the genetic diversity was 0.42. This level of population heterogeneity is indicative of the success of the California condor captive recovery program. We also suggested that among these 17 microsatellite loci, there could be a linkage between loci *D10* and *D6* (LOD score = 21.67), as well as between *A20* and *D9* (LOD score = 5.12), and between *B7* and *H238* (LOD score = 5.12) [10]. Continued effort in generating the condor linkage map is necessary to enhance genetic and genomic studies in this endemic New World vulture, including the linkage mapping of the dangerous inherited condition of chondrodystrophy.

The condor genome research project capitalizes on the current progress in avian comparative genomics studies, with the chicken genome sequence being the reference sequence for all other avian genomes. After establishing three potential linkages between 17 microsatellite loci, we undertook further steps to determine a larger set of genetically linked polymorphic markers and construct a preliminary genetic map for the California condor. The present investigation is aimed at developing a first-generation linkage map for this endangered New World vulture species. Here, we report the preliminary assessment of the large-scale condor genotyping data using an initial set of nearly 300 microsatellite markers and identifying linkage relationships between the validated polymorphic loci.

## 2. Materials and Methods

### 2.1. Samples

In order to genotype the resource population, we used DNAs isolated from the biosamples of 121 condor individuals that were stored at the Conservation Genetics facilities, Beckman Center for Conservation Research, San Diego Zoo Wildlife Alliance, and were previously tested with the 17 microsatellite markers [10,11]. The full list of these individuals is given in Supplementary Information (SI) S1, Table S1-2. The resource population included three samples for the known chondrodystrophic chicks: #160, #1405, and #2537. The DNA samples were kept at 4 °C for further amplification in polymerase chain reactions (PCR).

### 2.2. Marker Design

The above set of 17 microsatellite loci was developed by Genetic Identification Services (GIS), Chatsworth, CA [9,10]. Additionally, 929 clones representing the original microsatellite-enriched library generated by GIS were sequenced [9,10,11]. All microsatellite clone sequences have been deposited in the GenBank (Accession Numbers DQ471953, DQ483109–DQ484036, EF108178–EF108181, and EF116886–EF116903). Using these clone sequences and the Primer3 program available freely online elsewhere [26], we designed and tested almost 300 more new markers (see SI S2).

To amend the number of applicable polymorphic loci, we included three more avian microsatellite markers, *FhU2*, *HrU2*, and *HrU6* [27,28] known to be amplifiable and variable among many families of birds, including some New World vultures.

Overall, 316 loci (SI S3) were selected within the genotyping project including the following sets of markers: 296 newly designed loci (SI S2), 17 GIS loci [9,10], and 3 markers developed by Ellegren [27] and Primmer et al. [28].

The large-scale genotyping phase of the project included two phases, marker validation and genotyping itself, and was carried out at the UCLA Sequencing and Genotyping Core (see SI S1).

### 2.3. Marker Validation

To validate microsatellite markers, we used standard two-primer PCR amplification on a panel of eight individual samples as shown in Table 1.

For the amplification of few loci, we also utilized a cost-effective approach proposed by [29] that involved the fluorescent labeling of PCR fragments with the M13(-21) universal primer. The sequences of all forward primers were extended by incorporating the M13(-21) reverse compliment. Each reaction included two specific forward and reverse primers, and a third fluorescently labeled universal primer. Using this technique, we successfully produced the condor *FhU2* and *HrU2* PCR fragments, whereas *HrU6* failed in our hands [10].

The validation process relied on the following procedure. If the markers were amplifiable using the above-mentioned panel of DNA samples and PCR primers specific for a given marker and were polymorphic (with number of alleles ≥ 2), they were further employed for testing in the whole condor resource population.

### 2.4. Genotyping and Linkage Analysis

As a next step, we completed the two-point linkage analysis with software packages Locusmap [30] and CRIMAP [31], identified probable linkage groups (LGs) using the LOD value, and tested the marker order. Moreover, the probability of linking the chondrodystrophy trait to any LG was examined. The resulting linkage map was visualized using the MG2C online tool [32].

## 3. Results and Discussion

### 3.1. Marker Selection, Validation and Genotyping

Around 100 markers failed completely at the validation or genotyping phases, with no fragments amplified for any individual. The remaining 195 markers (SI S4) were successful, but there were 72 monomorphic loci that had no variation at all. In these cases, all individuals were homozygous for the same particular allele, and such markers did not undergo further analysis.

The distribution of the remaining 123 polymorphic loci for the observed number of alleles was as follows: 2 alleles, 80 loci; 3 alleles, 27 loci; 4 alleles, 9 loci; 5 alleles, 4 loci; 6, 7, or 8 alleles, 1 locus. The average number of alleles at these 123 loci was 2.6 per locus, i.e., close to the previous estimate in the study using 17 markers (2.41; [10]).

### 3.2. Linkage Analysis

In order to perform the two-point linkage analysis (SI S5), the 123 anonymous polymorphic microsatellite loci were used, while a total of 77 markers that were monomorphic and/or had no informative meioses were removed.

**Figure 1:**
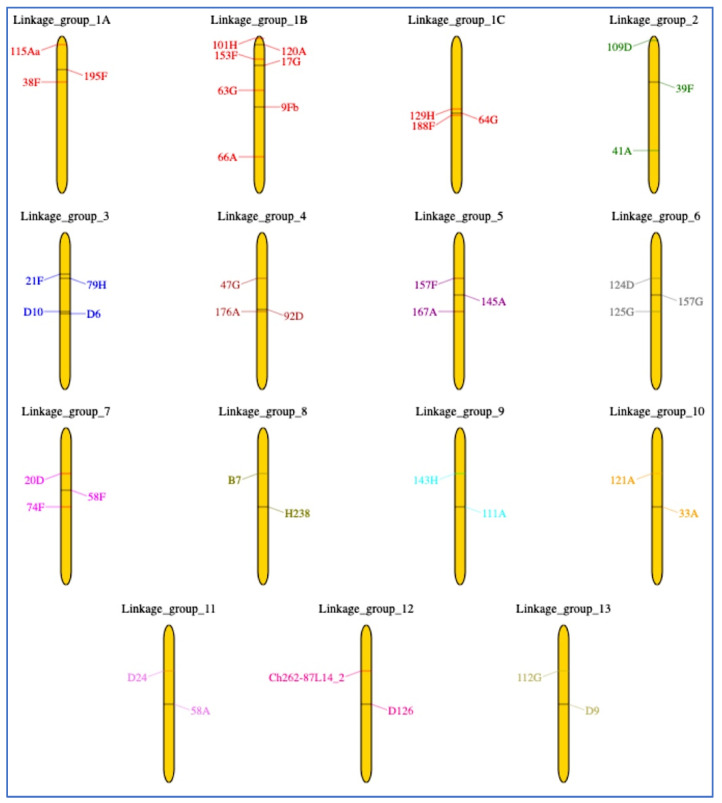
A first-generation California condor linkage map built using microsatellite loci. Length of linkage groups (LGs) and position of the loci are provisional, with a strong evidence of marker order being only available for subgroup 1A. Markers on the same LG are shown with the same colors.

The preliminary assessment of the available genotyping data suggested that we might expect to find about 10 or more LGs (LOD score ≥ 3). A refined linkage analysis (SI S6) revealed 15 LGs/subgroups that displayed varying degrees of the established marker ordering (Figure 1). LG 1 included subgroups 1A, 1B, and 1C. Subgroup 1A had strong evidence of the marker order, while 1B did not have strong ordering evidence, and 1C had no ordering information within this subgroup. Subgroups 1A and 1B could not be ordered relative to each other, and 1C displayed conflicting evidence concerning its linkage to 1A or 1B. Three 1C markers, 129H, 64G, and 188F, could be the same locus, but showed conflicting evidence about their linkages to subgroups 1A or 1B. LGs 3 to 7 possessed three linked markers each, and LGs 8 to 13 had only two linked markers each, although no marker ordering information was obtained for all those LGs.

In the present study, we confirmed the preliminary data of a tight linkage between the microsatellite loci *D10* and *D6* (LOD score = 21.67), and between *B7* and *H238* (LOD score = 5.12) [10]. The former pair of linked markers was localized here on LG 3, and the latter one on LG 8 (Figure 1). On the other hand, we did not see the previously suggested linkage between *A20* and *D9* (LOD score = 5.12) [10], although *D9* was linked to another marker, *112G*, forming LG 13 in this study. The inclusion of the *A20* locus into this LG will require further investigation.

As a result of the current genetic linkage analysis, we were unable to link the chondrodystrophy trait to any LG. The published genome assembly of two California condor birds [20] will allow for some comparison of the linkage map presented and the whole genome assemblies. In future studies, a greater effort is planned to essentially expand the linkage map and align the microsatellites used with the genome sequence. With the linkage map and condor population pedigree at hand, as well the DNA samples available for pedigreed chondrodystrophic and healthy individuals, it will be possible to use these novel microsatellites to conduct further linkage studies, find markers linked to chondrodystrophy or other important traits, and identify the causative gene mutations or other genome variants behind the inherited conditions in the California condor.

### 3.3. Polymorphic Marker Selection for Kinship Analyses

As a result of this study, we produced the condor microsatellite variation data that can be used to select polymorphic markers for a variety of genetic studies. For example, they would be good enough for paternity (parentage) analysis cases for chicks from condors living now in the wild. The condor rescue and propagation project will greatly benefit from genetic management based on this type of analysis using polymorphic microsatellite loci. For this purpose, a set of 18 markers was selected using the criteria of amplification reproducibility and locus polymorphism (with two and more alleles per locus), as seen in Table 2. This list can be amended further with more polymorphic markers, with the number of alleles varying from three to eight per locus.

Out of the above 18 polymorphic microsatellite loci, six were successfully implemented as a part of the test panel for the condor kinship analysis by Moran et al. [15], while seven were mapped to different LGs in the present investigation (Table 2). Moreover, a total of six markers (109D, 125G, D6, D9, D24, and D126) used in [15] were assigned to the linkage map here.

## 4. Conclusions and Expectations

In the present study as a first phase of the California condor genotyping and linkage mapping project, the resource population of 121 related birds was selected. The combined set of microsatellite markers contained 316 loci including 296 newly designed markers. Of these, 94 loci did not come through the validation phase and 27 more loci failed at the stage of actual genotyping. The remaining 195 filtered, validated, and amplifiable loci included 72 monomorphic markers and 123 polymorphic ones, with 1 to 121 individuals genotyped per locus and with the observed number of alleles ranging between 2 and 8. A panel of polymorphic microsatellite markers was selected and used for performing the paternity (parentage) analysis in chicks hatched from the eggs laid by condors in the wild [15]. This also aided in identifying a few cases of facultative parthenogenesis in California condors, being the first molecular marker-based detection of this asexual reproduction phenomenon in a bird species [16].

The data produced in the course of the genotyping project will be further used for estimating genetic statistics (heterozygosity, Hardy–Weinberg equilibrium, etc.) in the current population. The previous estimates demonstrated a sustained level of genetic diversity, whereas Hardy–Weinberg equilibrium deviations suggested a possible inbreeding impact in the condor population. These population genetic estimates facilitate the genetic management program for this endangered species, allowing for efforts that aim “to maximize the retention of genetic diversity by minimizing mean kinship within the population” [10].

The dataset of condor genotypes at informative polymorphic loci will also be utilized for the future genetic linkage analysis, with a hope to produce an updated genetic linkage map for condors as a helpful tool for locating and characterizing the candidate loci for hereditary conditions. An ultimate goal for further condor conservation genomics research will be the integration of the genetic linkage, and the cytogenetic and BAC-based physical maps, and the incorporation of this integral mapping information into the first-generation genome sequence [20,25] to improve its assembly and annotation. This will verify and rectify the condor assembled chromosome sequences for possible errors, aid in the assembly of missing microchromosomes, and help to reduce the number and size of unplaced/unlocalized scaffolds.

## Figures and Tables

**Table 1:** A panel of eight individual samples utilized for validating the microsatellite markers.

Chick #	Father #	Mother #	Sex	Chondrodystrophy Carrier
4	unknown	unknown	Male	No
10	unknown	unknown	Female	No
11	unknown	unknown	Female	No
12	unknown	unknown	Female	No
13	unknown	unknown	Female	No
20	unknown	unknown	Male	No
21	2	11	Male	No
25	3	12	Male	No

**Table 2:** A set of 18 polymorphic microsatellite markers chosen for paternity (parentage) analysis.

Marker	Forward Primer	Reverse Primer	No. of Alleles
*151F* *	GCTTCTCCAGAGAGCTCCAA	GCTCTTCAGCAGCTTTTGCT	5
*103D*	CCCATGGAATGGGAAAATAA	CATTTGCATCATGCTCAGGT	4
*133H* *	CAGAAATGCGCTTTGTGTGT	GCCTGTTGGGATGACTCCTA	4
*100A*	GTCATCCTCCTCCCTTCCTC	CCAGCATCATCAGTCACGTC	3
*144A* *	TATCGGAGGGCAGAGGACTA	TGCCTTCACTACTAAATATGGCTTT	3
*156A* *	CATTTCGTGGAAGCCAAAAC	TCCTTTCCCTACAGCCCTTT	3
*109D* *^†^	CGTGTCCTGCTGCATCTAAC	GAGGGAGAAAACAGGCAGTG	3
*125G* *^†^	GCCTATCATTTAGGCACAGAGA	GCCTGGGTATTCAGATGGAA	3
*101H* ^†^	CGTGTACACCTGCCTTTCCT	ATGGAGAGATGGGATGCAAG	3
*132H*	GAGCTTTCCAGACGTTGAGG	GATGCAAGAAAAGCGACACA	3
*66A* ^†^	AAAGGTGCGTGGTTCTGG	CTGGGGTCACAAAGAGGTTC	2
*98A*	TGGCACTGTGACTAAAGCAAA	TGAAAGGCAGTCAGCAGAGA	2
*135A*	CCCAAAAACTGATGAACAACG	ACAGGACCTTCTATGCCAAA	2
*9Fb* ^†^	TCGCCTTTTACTGCTGACTTC	AAGAGGAGGAGAGGCTACACG	2
*195F*	AACCTGGGTTTGAGTCATCG	ATGGTGCTGTGAAACTGTGC	2
*129H* ^†^	TCCTTGCTGGACTGACCTCT	AACTGGTCCGTCGATAGTGG	2
*CH262-13G5_1*	GTTCGTCCCCCTCATTTCTT	GGCGGCTTAGATGTGCAG	2
*CH262-87L14_2* ^†^	TCTTCTGCATCGCTGTGTTC	TCCCTGTCAGCTTACACTGCT	2

* Used for kinship analysis in [15]. ^†^ Mapped to LGs in this study.

## Data Availability

The data presented in this study are available in this article and supplementary material.

## Supplementary Materials

The following supporting information can be downloaded at: https://www.mdpi.com/article/10.3390/ani12233266/s1, Supplementary Information (SI) S1: Marker validation and genotyping in California condors; SI S2: Microsatellite marker and primer information for 296 loci; SI S3: Microsatellite marker and primer specifications for 316 loci; SI S4: Genotyping data for 195 loci; SI S5: Two-point linkage analysis results; SI S6: Genetic linkage analysis in California condors.
