# Supplementary material for: Towards a Genetic Linkage Map of the California Condor, an Endangered New World Vulture Species
Source: Animals (Basel). 2022 Nov 24;12(23):3266. doi: 10.3390/ani12233266 (PMC9739316; doi:10.3390/ani12233266)
Supplement: Supplementary file 1 [file animals-12-03266-s001.zip › Supplementary Information S1 revised.pdf]

Supplementary Information S1: Marker validation and genotyping in California condors

1. Validation phase

To validate a set of designed and selected condor microsatellite markers, a panel of eight individuals was used (Table 1).

At the validation phase, 94 microsatellite loci failed (Table S1-1) and were not used for further genotyping.

Table S1-1. Microsatellite markers that failed at the validation step or in the course of actual genotyping.

Non-validated (94 loci)	Failed Markers (27 loci)
2A, 8A, 42A, 44A, 48A, 67A, 107A, 112A, 117A, 119A, 133A, 134Aa, 138A, 155A, 172A, 4D, 10Db, 14D, 30Da, 35D, 43D, 46D, 50D, 55D, 61D, 84Da, 84Db, 102D, 118D, 133D, 9Fa, 36F, 46F, 53F, 63F/CH262-21P20, 76F, 84F, 96F, 104F, 116F, 117F, 163Fb, 172F, 180F, 181F, 184F, 185F, 190F, 201F, 205F, 207F, 216F, 241F, 257Fb, 13G, 18Ga, 18Gb, 18Gc, 93G, 114Ga, 114Gb, 144G, 148G, 151Ga, 151Gb, 160Ga, 160Gc, 165G, 173G, 179G, 199G, 200G, 201G, 7H, 29H, 60Ha, 61H, 71Ha, 86H, 114Ha, 114Hb, 119H, 128H, 137Ha, 137Hb, 140H, GIS8, GIS14, GIS18a, GIS18b, CH262-184F6_2, FhU2, HrU2, HrU6	*6A, 53D, 42F, 57F, 142F, 158F, 234F, 244F, 253Fa, 25Gb, 147G, 160Gb, 52H, 60Hb, 71Hb, 89H, 110Hb, 113H, 142Hb, CH262-181F1_1*

2. Genotyping phase

The list of all microsatellite markers tested is shown in Supplementary Information (SI) S4. Although there was a sufficient amount of DNA per sample for both the marker validation and genotyping steps, a preliminary whole genome amplification procedure was also used. However, in some cases there was not enough DNA to complete the project as planned.

2.1. Original (raw) datasets

In the course of the condor genotyping project reported here, we obtained from the UCLA Sequencing and Genotyping Core an enormous amount of raw data that were the machine-generated files, each containing multiple long strings of specific sample-marker combinations as shown in an example for one sample–marker combination (Figure S1-1).

```
Sample File → Sample Name → Run Name → Panel → Marker → Dye → Allele 1 → Size 1 → Height 1 → Allele 2 → Size 2 → Height 2 →
Allele 3 → Size 3 → Height 3 → Allele 4 → Size 4 → Height 4 → Allele 5 → Size 5 → Height 5 → Allele 6 → Size 6 → Height 6 →
Allele 7 → Size 7 → Height 7 → Allele 8 → Size 8 → Height 8 → Allele 9 → Size 9 → Height 9 → Allele 10 → Size 10 → Height 10 →
Allele 11 → Size 11 → Height 11 → Allele 12 → Size 12 → Height 12 → Allele 13 → Size 13 → Height 13 → Allele 14 → Size 14 →
Height 14 → Allele 15 → Size 15 → Height 15 → Allele 16 → Size 16 → Height 16 → Allele 17 → Size 17 → Height 17 → Allele 18 →
Size 18 → Height 18 → Allele 19 → Size 19 → Height 19 → Allele 20 → Size 20 → Height 20 → Allele 21 → Size 21 → Height 21 →
Allele 22 → Size 22 → Height 22 → Allele 23 → Size 23 → Height 23 → Allele 24 → Size 24 → Height 24 → Allele 25 → Size 25 →
Height 25 → Allele 26 → Size 26 → Height 26 → Allele 27 → Size 27 → Height 27 → Allele 28 → Size 28 → Height 28 → Allele 29 →
Size 29 → Height 29 → Allele 30 → Size 30 → Height 30 → Allele 31 → Size 31 → Height 31 → Allele 32 → Size 32 → Height 32 →
Allele 33 → Size 33 → Height 33 → Allele 34 → Size 34 → Height 34 → Allele 35 → Size 35 → Height 35 → Allele 36 → Size 36 →
Height 36 → Allele 37 → Size 37 → Height 37 → Allele 38 → Size 38 → Height 38 → Allele 39 → Size 39 → Height 39 → Allele 40 →
Size 40 → Height 40 → Allele 41 → Size 41 → Height 41 → Allele 42 → Size 42 → Height 42 → Allele 43 → Size 43 → Height 43 →
Allele 44 → Size 44 → Height 44 → Allele 45 → Size 45 → Height 45 → Allele 46 → Size 46 → Height 46 → Allele 47 → Size 47 →
Height 47 → Allele 48 → Size 48 → Height 48 → Allele 49 → Size 49 → Height 49 → Allele 50 → Size 50 → Height 50 → Allele 51 →
Size 51 → Height 51 → Allele 52 → Size 52 → Height 52 → Allele 53 → Size 53 → Height 53 → Allele 54 → Size 54 → Height 54 →
Allele 55 → Size 55 → Height 55 → Allele 56 → Size 56 → Height 56 → Allele 57 → Size 57 → Height 57 → Allele 58 → Size 58 →
Height 58 → Allele 59 → Size 59 → Height 59 → Allele 60 → Size 60 → Height 60 → Allele 61 → Size 61 → Height 61 → Allele 62 →
Size 62 → Height 62 → Allele 63 → Size 63 → Height 63 → Allele 64 → Size 64 → Height 64 → Allele 65 → Size 65 → Height 65 →
Allele 66 → Size 66 → Height 66 → Allele 67 → Size 67 → Height 67 → Allele 68 → Size 68 → Height 68 → Allele 69 → Size 69 →
Height 69 → Allele 70 → Size 70 → Height 70 → Allele 71 → Size 71 → Height 71 → Allele 72 → Size 72 → Height 72 → Allele 73 →
Size 73 → Height 73 → Allele 74 → Size 74 → Height 74 → Allele 75 → Size 75 → Height 75 → Allele 76 → Size 76 → Height 76 →
Allele 77 → Size 77 → Height 77 → Allele 78 → Size 78 → Height 78 → Allele 79 → Size 79 → Height 79 → Allele 80 → Size 80 →
Height 80 → Allele 81 → Size 81 → Height 81 → Allele 82 → Size 82 → Height 82 → Allele 83 → Size 83 → Height 83 → Allele 84 →
Size 84 → Height 84 → Allele 85 → Size 85 → Height 85 → Allele 86 → Size 86 → Height 86 → Allele 87 → Size 87 → Height 87 →
Allele 88 → Size 88 → Height 88 → Allele 89 → Size 89 → Height 89 → Allele 90 → Size 90 → Height 90 → Allele 91 → Size 91 →
Height 91 → Allele 92 → Size 92 → Height 92 → Allele 93 → Size 93 → Height 93 → Allele 94 → Size 94 → Height 94 → Allele 95 →
Size 95 → Height 95 → Allele 96 → Size 96 → Height 96 → Allele 97 → Size 97 → Height 97 → Allele 98 → Size 98 → Height 98 →
Allele 99 → Size 99 → Height 99 → Allele 100 → Size 100 → Height 100 → Allele 101 → Size 101 → Height 101 → Allele 102 → Size
102 → Height 102 → Allele 103 → Size 103 → Height 103 → Allele 104 → Size 104 → Height 104 → Allele 105 → Size 105 → Height 105
→ Allele 106 → Size 106 → Height 106 → Allele 107 → Size 107 → Height 107 → Allele 108 → Size 108 → Height 108 → Allele 109 →
Size 109 → Height 109 → Allele 110 → Size 110 → Height 110 → Allele 111 → Size 111 → Height 111 → Allele 112 → Size 112 →
Height 112 → Allele 113 → Size 113 → Height 113 → Allele 114 → Size 114 → Height 114 → Allele 115 → Size 115 → Height 115 →
Allele 116 → Size 116 → Height 116 → Allele 117 → Size 117 → Height 117 → Allele 118 → Size 118 → Height 118 → Allele 119 →
Size 119 → Height 119 → Allele 120 → Size 120 → Height 120 → Allele 121 → Size 121 → Height 121 → Allele 122 → Size 122 →
Height 122 → Allele 123 → Size 123 → Height 123 → Allele 124 → Size 124 → Height 124 → Allele 125 → Size 125 → Height 125 →
Allele 126 → Size 126 → Height 126 → Allele 127 → Size 127 → Height 127 → Allele 128 → Size 128 → Height 128 → Allele 129 →
Size 129 → Height 129 → Allele 130 → Size 130 → Height 130 → Allele 131 → Size 131 → Height 131 → Allele 132 → Size 132 →
Height 132 → Allele 133 → Size 133 → Height 133 → Allele 134 → Size 134 → Height 134 → Allele 135 → Size 135 → Height 135 →
Allele 136 → Size 136 → Height 136 → Allele 137 → Size 137 → Height 137 → Allele 138 → Size 138 → Height 138 → Allele 139 →
Size 139 → Height 139 → Allele 140 → Size 140 → Height 140 → Allele 141 → Size 141 → Height 141 → Allele 142 → Size 142 →
```

```
Height 142 → Allele 143 → Size 143 → Height 143 → Allele 144 → Size 144 → Height 144 → Allele 145 → Size 145 → Height 145 →
Allele 146 → Size 146 → Height 146 → Allele 147 → Size 147 → Height 147 → Allele 148 → Size 148 → Height 148 → Allele 149 →
Size 149 → Height 149 → Allele 150 → Size 150 → Height 150 → Allele 151 → Size 151 → Height 151 → Allele 152 → Size 152 →
Height 152 → Allele 153 → Size 153 → Height 153 → Allele 154 → Size 154 → Height 154 → Allele 155 → Size 155 → Height 155 →
Allele 156 → Size 156 → Height 156 → Allele 157 → Size 157 → Height 157 → Allele 158 → Size 158 → Height 158 → Allele 159 →
Size 159 → Height 159 → Allele 160 → Size 160 → Height 160 → Allele 161 → Size 161 → Height 161 → Allele 162 → Size 162 →
Height 162 → Allele 163 → Size 163 → Height 163 → Allele 164 → Size 164 → Height 164 → Allele 165 → Size 165 → Height 165 →
Allele 166 → Size 166 → Height 166 → Allele 167 → Size 167 → Height 167 → Allele 168 → Size 168 → Height 168 → Allele 169 →
Size 169 → Height 169 → Allele 170 → Size 170 → Height 170 → Allele 171 → Size 171 → Height 171 → Allele 172 → Size 172 →
Height 172 → Allele 173 → Size 173 → Height 173 → Allele 174 → Size 174 → Height 174 → Allele 175 → Size 175 → Height 175 →
Allele 176 → Size 176 → Height 176 → Allele 177 → Size 177 → Height 177 → Allele 178 → Size 178 → Height 178 → Allele 179 →
Size 179 → Height 179 → Allele 180 → Size 180 → Height 180 → Allele 181 → Size 181 → Height 181 → Allele 182 → Size 182 →
Height 182 → Allele 183 → Size 183 → Height 183 → Allele 184 → Size 184 → Height 184 → Allele 185 → Size 185 → Height 185 →
Allele 186 → Size 186 → Height 186 → Allele 187 → Size 187 → Height 187 → Allele 188 → Size 188 → Height 188 → Allele 189 →
Size 189 → Height 189 → Allele 190 → Size 190 → Height 190 → Allele 191 → Size 191 → Height 191 → Allele 192 → Size 192 →
Height 192 → Allele 193 → Size 193 → Height 193 → Allele 194 → Size 194 → Height 194 → Allele 195 → Size 195 → Height 195 →
Allele 196 → Size 196 → Height 196 → Allele 197 → Size 197 → Height 197 → Allele 198 → Size 198 → Height 198 → Allele 199 →
Size 199 → Height 199 → Allele 200 → Size 200 → Height 200 → Allele 201 → Size 201 → Height 201 → Allele 202 → Size 202 →
Height 202 → Allele 203 → Size 203 → Height 203 → Allele 204 → Size 204 → Height 204 → Allele 205 → Size 205 → Height 205 →
Allele 206 → Size 206 → Height 206 → Allele 207 → Size 207 → Height 207 → Allele 208 → Size 208 → Height 208 → Allele 209 →
Size 209 → Height 209 → Allele 210 → Size 210 → Height 210 → Allele 211 → Size 211 → Height 211 → Allele 212 → Size 212 →
Height 212 → Allele 213 → Size 213 → Height 213 → Allele 214 → Size 214 → Height 214 → Allele 215 → Size 215 → Height 215 →
Allele 216 → Size 216 → Height 216 → Allele 217 → Size 217 → Height 217 → Allele 218 → Size 218 → Height 218 → Allele 219 →
Size 219 → Height 219 → Allele 220 → Size 220 → Height 220 → Allele 221 → Size 221 → Height 221 → Allele 222 → Size 222 →
Height 222 → Allele 223 → Size 223 → Height 223 → Allele 224 → Size 224 → Height 224 → Allele 225 → Size 225 → Height 225 →
Allele 226 → Size 226 → Height 226 → Allele 227 → Size 227 → Height 227 → Allele 228 → Size 228 → Height 228 → Allele 229 →
Size 229 → Height 229 → Allele 230 → Size 230 → Height 230 → Allele 231 → Size 231 → Height 231 → Allele 232 → Size 232 →
Height 232 → Allele 233 → Size 233 → Height 233 → Allele 234 → Size 234 → Height 234 → Allele 235 → Size 235 → Height 235 →
Allele 236 → Size 236 → Height 236 → Allele 237 → Size 237 → Height 237 → Allele 238 → Size 238 → Height 238 → Allele 239 →
Size 239 → Height 239 → Allele 240 → Size 240 → Height 240 → Allele 241 → Size 241 → Height 241 → Allele 242 → Size 242 →
Height 242 → Allele 243 → Size 243 → Height 243 → Allele 244 → Size 244 → Height 244 → Allele 245 → Size 245 → Height 245 →
Allele 246 → Size 246 → Height 246 → Allele 247 → Size 247 → Height 247 → Allele 248 → Size 248 → Height 248 → Allele 249 →
Size 249 → Height 249 → Allele 250 → Size 250 → Height 250 → Allele 251 → Size 251 → Height 251 → Allele 252 → Size 252 →
Height 252 → Allele 253 → Size 253 → Height 253 → Allele 254 → Size 254 → Height 254 → Allele 255 → Size 255 → Height 255 →
Allele 256 → Size 256 → Height 256 → Allele 257 → Size 257 → Height 257 → Allele 258 → Size 258 → Height 258 → Allele 259 →
Size 259 → Height 259 → Allele 260 → Size 260 → Height 260 → Allele 261 → Size 261 → Height 261 → Allele 262 → Size 262 →
Height 262 → Allele 263 → Size 263 → Height 263 → Allele 264 → Size 264 → Height 264 → Allele 265 → Size 265 → Height 265 →
Allele 266 → Size 266 → Height 266 → Allele 267 → Size 267 → Height 267 → Allele 268 → Size 268 → Height 268 → Allele 269 →
Size 269 → Height 269 → Allele 270 → Size 270 → Height 270 → Allele 271 → Size 271 → Height 271 → OS → AN → GQ →
Con01-9Fb-86F-192F-244F_196_F11.fsa → 196 → Con01-9Fb-86F-192F-244F_2011-02-16_1 → 9Fb-86F-192F-244F → 192F → Y → 309 → 309.01
→ 194 → 311 → 311.17 → 353 → → → → → → → → → → → → → → → → → → → → → → → → → → → → → → → → →
→ → → → → → → → → → → → → → → → → → → → → → → → → → → → → → → → → → → → → → → → → → → →
→ → → → → → → → → → → → → → → → → → → → → → → → → → → → → → → → → → → → → → → → → → → →
→ → → → → → → → → → → → → → → → → → → → → → → → → → → → → → → → → → → → → → → → → → → →
→ → → → → → → → → → → → → → → → → → → → → → → → → → → → → → → → → → → → → → → → → → → →
→ → → → → → → → → → → → → → → → → → → → → → → → → → → → → → → → → → → → → → → → → → → →
→ → → → → → → → → → → → → → → → → → → → → → → → → → → → → → → → → → → → → → → → → → → →
→ → → → → → → → → → → → → → → → → → → → → → → → → → → → → → → → → → → → → → → → → → → →
→ → → → → → → → → → → → → → → → → → → → → → → → → → → → → → → → → → → → → → → → → → → →
→ → → → → → → → → → → → → → → → → → → → → → → → → → → → → → → → → → → → → → → → → → → →
→ → → → → → → → → → → → → → → → → → → → → → → → → → → → → → → → → → → → → → → → → → → →
→ → → → → → → → → → → → → → → → → → → → → → → → → → → → → → → → → → → → → → → → → → → →
→ → → → → → → → → → → → → → → → → → → → → → → → → → → → → → → → → → → → → → → → → → → →
→ → → → → → → → → → → → → → → → → → → → → → → → → → → → → → → → → → → → → → → → → → → →
→ → → → → → → → → → → → → → → → → → → → → → → → → → → → → → → → → → → → → → → → → → → →
→ → → → → → → → → → → → → → → → → → → → → → → → → → → → → → → → → → → → → → → → → → → →
→ → → → → → → → → → → → → → → → → → → → → → → → → → → → → → → → → → → → → → → → → → → →
→ → → → → → → → → → → → → → → → → → → → → → → → → → → → → → → → → → → → → → → → → → → →
-1.0 → -1.0 → 1.0 →
```

Figure S1-1. An example of a raw genotyping data file with initial lines for one sample-marker combination.

In all, there were 90 text files with a total of 22.217 MB raw data information that were subject to the initial review, processing, editing, correction and assessment. The analysis outline included the following steps:

(a) overall evaluation of genotyping data integrity and completeness,
(b) identifying missing data,
(c) preparation of a spreadsheet with the summarized genotyping data,
(d) estimation of effort required for constructing a linkage map,
(e) preparation of a report on the preliminary evaluation and analysis.

In practice, there were three rounds of data revision and filtering for incomplete and missing entries as will be outlined below.

2.2. First round

To generate the data, we had 121 condor individuals (Table S1-2) for genotyping with 296 new microsatellite markers (SI S3).

Table S1-2. A list of the 121 resource population individuals that were used as DNA source for genotyping.

Ordinal No.	Chick #	Dad #	Mom #	Sex	Chondrodystrophy (0, no; 1, yes)
**1**	4	0	0	M	0
**2**	10	0	0	F	0
**3**	11	0	0	F	0
**4**	12	0	0	F	0
**5**	13	0	0	F	0
**6**	20	0	0	M	0
**7**	21	2	11	M	0
**8**	23	4	8	M	0
**9**	25	3	12	M	0
**10**	27	4	8	M	0
**11**	30	3	12	F	0
**12**	31	3	12	F	0
**13**	32	6	10	F	0
**14**	37	6	10	F	0
**15**	39	3	12	F	0
**16**	40	3	12	F	0
**17**	42	6	10	M	0
**18**	44	21	12	M	0
**19**	45	20	13	F	0
**20**	47	20	13	M	0
**21**	48	27	31	M	0
**22**	54	42	39	F	0
**23**	55	27	31	M	0
**24**	56	27	31	F	0
**25**	57	23	32	F	0
**26**	59	42	39	F	0
**27**	64	21	40	F	0
**28**	75	27	31	M	0
**29**	76	27	31	F	0
**30**	77	27	31	F	0
**31**	78	21	40	M	0
**32**	79	21	40	F	0
**33**	81	42	39	F	0
**34**	82	23	32	F	0
**35**	83	25	37	M	0
**36**	84	25	37	F	0
**37**	87	23	32	M	0
**38**	88	23	32	F	0
**39**	89	27	31	M	0
**40**	92	42	39	M	0
**41**	97	44	45	F	0
**42**	99	27	31	M	0
**43**	100	25	37	M	0
**44**	101	25	37	F	0
**45**	102	23	32	M	0
**46**	103	23	32	F	0
**47**	107	21	40	M	0
**48**	108	42	39	F	0
**49**	111	44	45	F	0
**50**	113	44	45	F	0
**51**	114	23	32	M	0
**52**	122	21	40	M	0
**53**	123	42	39	M	0

**54**	124	42	39	F	0
**55**	125	44	45	M	0
**56**	126	44	45	F	0
**57**	127	25	37	F	0
**58**	128	27	31	F	0
**59**	142	27	31	M	0
**60**	143	27	31	F	0
**61**	145	42	39	M	0
**62**	146	42	39	F	0
**63**	149	21	40	F	0
**64**	150	47	30	F	0
**65**	151	21	40	F	0
**66**	152	25	37	F	0
**67**	154	44	45	F	0
**68**	156	21	40	F	0
**69**	**160**	**27**	**31**	**F**	**1**
**70**	161	25	37	F	0
**71**	162	42	39	M	0
**72**	164	23	32	M	0
**73**	165	47	30	M	0
**74**	169	23	32	M	0
**75**	170	42	39	M	0
**76**	174	44	45	F	0
**77**	175	25	37	M	0
**78**	180	44	45	F	0
**79**	182	47	30	F	0
**80**	183	23	32	F	0
**81**	185	42	39	M	0
**82**	189	23	32	M	0
**83**	191	47	30	F	0
**84**	192	21	40	F	0
**85**	195	44	45	F	0
**86**	196	25	37	F	0
**87**	203	47	30	M	0
**88**	210	47	30	F	0
**89**	212	21	40	F	0
**90**	213	25	37	M	0
**91**	214	23	32	F	0
**92**	217	44	45	F	0
**93**	219	44	45	M	0
**94**	222	25	37	F	0
**95**	225	23	32	F	0
**96**	231	21	40	F	0
**97**	235	47	30	F	0
**98**	236	23	32	F	0
**99**	237	44	45	M	0
**100**	238	25	37	M	0
**101**	241	55	84	F	0
**102**	242	44	45	M	0
**103**	243	47	30	M	0
**104**	250	55	84	M	0
**105**	251	21	40	M	0
**106**	257	47	30	M	0
**107**	264	25	37	F	0
**108**	265	44	45	M	0

**109**	269	42	39	M	0
**110**	270	44	45	M	0
**111**	272	55	84	M	0
**112**	280	47	30	F	0
**113**	286	23	32	M	0
**114**	292	42	39	F	0
**115**	297	47	30	F	0
**116**	309	47	30	M	0
**117**	313	42	39	M	0
**118**	332	42	39	M	0
**119**	341	55	84	M	0
**120**	**1405**	**27**	**31**	**M**	**1**
**121**	**2537**	**27**	**31**	**M**	**1**

The loci that were supposed to be used in the post-genotyping analysis included the following combined set of markers:

Newly designed:	296 markers
Old:	17 original + 3 Ellegren's markers
Total:	316

If all markers would be workable in all 121 individuals, we would expect a total of 38,236 genotypes.

There were various errors in the data that required a very careful check of almost every datapoint out of approximately 25,000 genotyping entries that remained after removing the failed markers.

2.3. Second round

At this step, we looked more deeply into the datasets produced for individual markers by sifting an estimate of 25,000 datapoints. The actual data contained entries for 198 loci, and if we had 100% success rate for all of them, the expected total number of genotypes in 121 individuals would be 23,958.

In reality, there were entries with "zero" alleles (i.e. unknown or missed genotypes) for certain bird-marker combinations. Because of that, we deleted more than 4,100 sample-locus combinations. In addition to the markers that initially failed at the validation step, we found a good number of 28 microsatellite markers that were used for genotyping but failed in all individuals. We deleted such entries from the entire dataset. After that, we had about 17,500 complete entries with both alleles available that were further used for the analysis within the condor genotyping project.

For several loci, our UCLA collaborators produced duplicated genotypes, probably because the initial genotyping did not result in convincing allele calls or amplification failed at all or because of improper handling of the genotyping data. For example, the original files contained duplicated genotyping data for the following loci:

121A	*135A*	*21A*
129H	*165A*	*5A*
130G	*18H*	*68A*

After removing all duplicated and failed genotypes, the data included 17,199 entries.

2.4. Third round

This round of the data processing involved a thorough double check of the entries for all possible errors. As a result, we obtained 17,178 datapoints for genotypes at 195 loci (SI S5). In addition, there were 121 loci excluded from the further analysis because these microsatellite markers failed at the validation step or in the course of actual genotyping. Table S1-1 shows the lists of such markers.

The previous estimate of 28 failed loci was corrected and reduced to 27 because one marker, *236G*, was mislabeled and it was actually *236F* that did not fail. There were few more examples of marker mislabeling when *23b* should be *23Fb*, *115A* should be *115Aa*, and *G1S17* should be *GIS17*. One individual, #254, occurred 180 times in the dataset, although it was not included in the original genotyping population of 121 birds. Because there is no #264 in the data meaning that # 254 is a sample mislabeling case, #254 was changed for #264.

At few loci, there was just few entries obtained. For instance, at *130G* locus the genotyping of all but one individual, #213, failed. For *56D* locus, there were only four samples available.

The number of valid datapoints would probably be corrected, i.e. reduced, if some entries for descendants from certain parents have alleles that were miscalled or contained other errors and are not expected judging from the parental genotypes. Examples of such situations are described below.

2.5. Missing and miscalled allele data

There were situations when one of two alleles at a locus was questionable and marked with "?". Parents/offspring genotypes were used to figure out such uncertain allele as it was determined for example, for the condor #4 at *148A* locus. However, it was sometimes impossible to identify an unknown allele as it was the case for #191 at *148A* locus.

There were entries marked with two question marks ("? ?") for both alleles at one locus and for one bird, for example, #161 at *60A* locus. Based on parental genotypes, we were able to determine that one of the alleles should be 179, while the other one could be 171 or 179 and was entered as 0. We had some other similar cases (e.g., #31 at *71Hb* locus).

In few cases, the duplicated individual-locus combinations did not show the same alleles and were resolved by looking at parents' or offspring's alleles at that locus. For instance, individual #4 at *135A* locus had two sets of data: 235/239 and 237/241. The #4 descendants, #23 and #27, had the 235/239 and 235/235 genotypes, respectively. This ruled out the 237/241 genotype for #4. Similar cases were: #103 at *129H* (255/369); #146 at *129H* (255/255); #183 at *129H* (255/369); and #196 at *129H* (255/255).

In some other cases, it was impossible to determine and confirm the right genotypes based on the closest relatives' genotypes, and they could doubtfully be used for the further analysis. For example, #196 at *135A* locus had 235/239 or 239/239. Its parents, #25 and 37, had respectively 235/239 and 239/239. In this case, the genotype for #196 was left as 0/239 where 0 means an uncertain allele. Similar cases were: #195 at *121A*; #203 at *129H*; #88 at *135A*; and #180 at *135A*. Such data were deleted in the whole dataset.

At *165A* locus, in addition to duplicated data, there was homozygosity, with only one allele, 189, present in the population. However, the "tail" of the samples, 235 to 2537, contained the 188 allele that looks improbable. They were changed for the 189 allele.

At *156A* locus, in addition to duplicated data for the samples 235 to 2537 that were genotyped for "*156a*", there was a similar shift in allele calling. Instead of 225, 229 and 237, they had the 226, 230 and 238 alleles.

This was corrected. For #292, two sets of alleles were produced, 229/229 and 229/237, but the parents genotypes did not allow to figure out the exact genotype of the descendant.

A similar but more complicated situation was observed and corrected, where possible, for *5A* locus (or "*5a*" as denoted in the duplicated cases) as can be seen in Table S1-3.

Table S1-3. An example of discrepancy in the allele calling data between chicks #235 to #2537 and their parents when genotyped for *5A* locus.

Chick #	Locus	Genotype	Parents	Corrected
**235**	*5A*	234/244	210/244 & 228/228	228/?
**236**	*5A*	234/244	210/236 & 228/244	236/244
**236**	*5a*	235/245		
**237**	*5A*	210/234	236/244 & 228/228	228/?
**237**	*5a*	249/249		
**238**	*5A*	210/210	210/228 & 210/228	210/210
**238**	*5a*	249/249		
**241**	*5A*	210/228	210/228 & 210/228	210/228
**241**	*5a*	249/249		
**242**	*5A*	210/244	236/244 & 228/228	228/?
**242**	*5a*	249/249		
**243**	*5A*	228/244	210/244 & 228/228	228/244
**243**	*5a*	249/249		
**250**	*5A*	228/228	210/228 & 210/228	228/228
**250**	*5a*	249/249		
**251**	*5A*	234/244	210/244 & 228/236	236/244
**251**	*5a*	235/245		
**257**	*5a*	227/245	210/244 & 228/228	228/244
**257**	*5A*	228/244		
**264**	*5a*	227/235	210/228 & 210/228	?
**265**	*5a*	227/247	236/244 & 228/228	228/244
**265**	*5A*	228/246		
**270**	*5a*	209/245	236/244 & 228/228	228/?
**270**	*5A*	210/244		
**272**	*5a*	209/209	210/228 & 210/228	210/210
**272**	*5A*	210/210		
**280**	*5A*	234/244	210/244 & 228/228	228/?
**280**	*5a*	235/245		
**286**	*5a*	227/235	210/236 & 228/244	228/236
**286**	*5A*	228/234		
**292**	*5A*	210/234	210/228 & 228/236	210/236
**292**	*5a*	235/235		
**297**	*5a*	209/227	210/244 & 228/228	210/228
**297**	*5A*	210/228		
**309**	*5a*	209/235	210/244 & 228/228	228/?
**309**	*5A*	210/234		
**313**	*5A*	228/234	210/228 & 228/236	228/236
**332**	*5a*	227/227	210/228 & 228/236	228/228
**332**	*5A*	228/228		
**341**	*5a*	209/227	210/228 & 210/228	210/228
**1405**	*5A*	210/228	210/236 & 228/236	210/228
**2537**	*5a*	227/235	210/236 & 228/236	228/236
**2537**	*5A*	228/234		

Overall, it was found that many loci had discrepancy and errors in allele calling for a subset of samples #235 to #2537 that is mentioned above as a "tail".

In many cases, the data were produced for individuals #4 to #231 or #235 to #2537 but not for both subsets. For example, the genotype entries were only available for samples #4 to #231 at *111A* locus, and for #235 to #2537 at loci *123A*, *131A* and *144A*.

In terms of data incompleteness, we observe a range of one to 121 genotyped samples per locus. This variation of incomplete data for 195 filtered, validated and amplified loci (markers) can be seen in Table S1-4.

Table S1-4. Numbers of condor individuals genotyped per locus.

Marker	Genotyped samples	Marker	Genotyped samples	Marker	Genotyped samples	Marker	Genotyped samples
130G	1	*58F*	74	*148A*	103	*176A*	111
56D	4	*48G*	75	*30H*	103	*42Da*	111
115F	18	*112G*	76	*5A*	103	*51H*	111
95A	18	*20F*	78	*125D*	104	*81H*	111
129A	19	*86F*	78	*168G*	104	*85F*	111
68F	21	*65F*	79	*175Ab*	104	*100A*	112
38H	21	*188F*	81	*64G*	105	*151F*	112
1A	22	*134Ab*	82	*163Fa*	105	*156A*	112
237F	22	*23b*	82	*180G*	105	*105A*	112
32F	22	*257Fa*	82	*109G*	105	*125A*	112
51G	22	*25Hb*	82	*109D*	106	*157G*	112
82D	22	*101H*	84	*153A*	106	*21D*	112
115Ab	22	*233F*	84	*165F*	106	*81G*	112
77A	23	*142Hc*	85	*220F*	106	*92D*	112
144A	24	*103D*	86	*252F*	106	*110Ha*	112
123A	24	*124D*	86	*41A*	106	*11A*	112
46A	24	*145A*	86	*53A*	106	*174A*	113
131A	25	*60A*	86	*CH262-184F1_2*	106	*42Db*	113
157A	25	*13Hc*	87	*CH262-53D16_2*	106	*CH262-13G5_2*	113
49D	27	*36D*	87	*115Aa*	106	*104D*	114
236F	36	*92H*	87	*133H*	107	*39D*	114
82G	45	*100F*	88	*116D*	107	*54G*	114
182G	46	*55A*	88	*143H*	107	*63G*	114
58D	50	*25Ha*	89	*30A*	107	*91A*	114
167A	52	*13Hb*	91	*68a*	107	*9A*	114
33G	53	*33A*	91	*73F*	107	*CH262-184F6_1*	114
21A	54	*57D*	91	*132H*	108	*110A*	114
97G	54	*111H*	91	*120H*	108	*17H*	115
150A	57	*124H*	92	*154A*	108	*38F*	115
153F	57	*111A*	92	*157F*	108	*47G*	115
A8	57	*27H*	93	*15A*	108	*58A*	115
C5	57	*82F*	93	*21F*	108	*80A*	115
G8	57	*253Fb*	94	*35F*	108	*135A*	116
H106	57	*57H*	94	*51A*	108	*37H*	116
H3	57	*CH262-53D16_1*	94	*13Ha*	109	*52A*	116
A20	58	*120A*	96	*198G*	109	*135H/CH262-154B5*	117
B7	58	*94G*	96	*25Ga*	109	*66A*	117
D10	58	*121H*	98	*35A*	109	*Ch262-87L14_2*	117
D126	58	*18H*	98	*37G*	109	*GIS17*	117
D24	58	*CH262-21P20_2*	98	*39F*	109	*CH262-87L14_1*	117
D6	58	*120D*	98	*74F*	109	*125G*	118
D9	58	*20D*	99	*116A*	110	*129H*	118
H115	58	*75H*	99	*139H*	110	*9Fb*	118
H127	58	*166F*	100	*186A*	110	*CH262-13G5_1*	118
H238	58	*192F*	100	*74H*	110	*111G*	119

H269	58	*121A*	100	*79H*	110	*165A*	119
H6	58	*56A*	101	*106D*	110	*195F*	121
17G	70	*195G*	102	*123H*	111	*98A*	121
142Ha	73	*63H*	102	*14A*	111		

2.6. Evaluation of monomorphic vs. polymorphic loci

The genotyping results showed that in the resource population of 121 condors, there were multiple monomorphic loci. To keep track of how many they are, we screened the total dataset for observed number of alleles per locus. These data are summarized in Table S1-5.

Table S1-5. Observed numbers of alleles per locus.

Marker	No. of alleles	Marker	No. of alleles	Marker	No. of alleles	Marker	No. of alleles
106D	1	*57H*	1	*15A*	2	*H106*	2
109G	1	*58D*	1	*167A*	2	*H115*	2
110Ha	1	*64G*	1	*174A*	2	*H127*	2
111G	1	*68a*	1	*17G*	2	*H238*	2
111H	1	*68F*	1	*180G*	2	*H3*	2
115Ab	1	*75H*	1	*192F*	2	*100A*	3
116A	1	*77A*	1	*195F*	2	*101H*	3
116D	1	*80A*	1	*1A*	2	*109D*	3
121H	1	*81G*	1	*21A*	2	*125G*	3
124H	1	*81H*	1	*21D*	2	*132H*	3
125D	1	*82D*	1	*21F*	2	*134Ab*	3
129A	1	*91A*	1	*220F*	2	*144A*	3
130G	1	*92H*	1	*233F*	2	*156A*	3
135H/CH262-154B5	1	*CH262-13G5_2*	1	*237F*	2	*157F*	3
139H	1	*CH262-184F1_2*	1	*23b*	2	*165F*	3
13Ha	1	*CH262-184F6_1*	1	*252F*	2	*166F*	3
13Hb	1	*CH262-21P20_2*	1	*27H*	2	*176A*	3
13Hc	1	*CH262-53D16_1*	1	*30A*	2	*20D*	3
142Ha	1	*CH262-53D16_2*	1	*33A*	2	*253Fb*	3
142Hc	1	*CH262-87L14_1*	1	*35A*	2	*32F*	3
150A	1	*GIS17*	1	*35F*	2	*38H*	3
153A	1	*188F*	1?	*36D*	2	*39F*	3
154A	1	*39D*	1 (2)	*37G*	2	*74H*	3
163Fa	1	*60A*	2?	*38F*	2	*79H*	3
165A	1	*100F*	2	*47G*	2	*85F*	3
168G	1	*104D*	2	*51H*	2	*94G*	3
175Ab	1	*105A*	2	*54G*	2	*97G*	3
17H	1	*110A*	2	*56A*	2	*C5*	3
182G	1	*111A*	2	*57D*	2	*D6*	3
18H	1	*112G*	2	*58A*	2	*D9*	3
195G	1	*115Aa*	2	*58F*	2	*H269*	3
198G	1	*115F*	2	*63G*	2	*H6*	3
20F	1	*11A*	2	*63H*	2	*103D*	4
236F	1	*120A*	2	*65F*	2	*121A*	4
257Fa	1	*120D*	2	*66A*	2	*133H*	4
25Ga	1	*120H*	2	*73F*	2	*186A*	4
25Ha	1	*123A*	2	*82F*	2	*41A*	4
25Hb	1	*123H*	2	*86F*	2	*46A*	4
30H	1	*124D*	2	*95A*	2	*5A*	4

33G	1	*125A*	2	*98A*	2	*92D*	4
37H	1	*129H*	2	*9Fb*	2	*A8*	4
42Da	1	*131A*	2	*A20*	2	*151F*	5
48G	1	*135A*	2	*B7*	2	*42Db*	5
51A	1	*143H*	2	*CH262-13G5_1*	2	*82G*	5
51G	1	*145A*	2	*CH262-87L14_2*	2	*49D*	5?
52A	1	*148A*	2	*D10*	2	*74F*	6
53A	1	*14A*	2	*D126*	2	*157G*	7
55A	1	*153F*	2	*D24*	2	*9A*	8
56D	1	*157A*	2	*G8*	2		

A total of 72 monomorphic loci were identified in this population. For the subsequent linkage analysis, they have no value and can be set aside.

3. Further planned steps

Because of the gaps in the data when we have zeros (i.e. unknown or missed genotypes) for certain bird-marker combinations, there is a need in analyzing such entries and attempting to identify likely genotypes for them based on known genotypes of their parents and/or descendants. We may proceed with that to get more rectified data with fewer gaps before doing next steps in the data analyses. Alternatively, we may just throw away such data. Additionally, there will be a need in identifying allele calling errors when genotypes of parents and offspring do not conform to each other.

The data obtained can be used in two ways–to analyze genetic variation within the current California condor population and to assess genetic linkage relationships between the markers used for genotyping.

3.1. Genetic variation analysis

On the basis of the genotypes produced for 195 microsatellite loci, we can evaluate genetic diversity, heterozygosity, Hardy–Weinberg equilibrium model and other standard population genetic parameters. There are two options to do this estimation:

(a) *Analysis of the identified full set of loci*

At the moment, we have the list of 195 loci filtered as described above, and the appropriate whole dataset may be used for population genetic analyses.

However, if we look at available population genetic analysis programs (e.g., here: http://www.biology.duke.edu/noorlab/software.htm), it would be quite complicated to find an appropriate one and use it to treat the whole dataset for determining certain population/variation statistics. It is doubtful that any of these programs could handle such a huge data array. Another problem is multiple gaps we have. We already shortened the dataset by removing zeros and some questionable entries. The available programs do require entering zeros in the case of gaps at any locus. That means that it would be necessary to revert the data back to the original form. It might not be that easy to manipulate with a set of 17 to 25 thousand entries back and forth.

Also, if we undertake the full dataset analysis, it is even hard to imagine the size of an input file and the length of each row in it for the case of almost 200 loci. It is probably impossible to prepare a spreadsheet in Excel for such a long row, with up to 400 cells (200 loci by 2 alleles per individual).

(b) Analysis of the data subset

As an alternative to the full dataset analysis, we may carry out a pilot analysis of approximately 20 loci. For this purpose, we would suggest the following. The plan was to genotype a resource population of 121 individuals for over 300 markers. About 100 markers were not validated and used for the actual genotyping. After removing additionally the failed markers, we still have a decent number of 195 genotyped loci that are either complete for every individual or incomplete to a different extent, which was observed in most cases. For population statistics, it seems reasonable to select randomly and use, say, 20 or so "good" loci with complete or mostly complete (e.g., $n \geq 110$ samples) genotyping data for the individuals studied. Moreover, such a reduced dataset would be better fitting the analyzing capacity of most known software programs, and it would be easier to handle it.

If we chose a subset of "good" and fairly complete data for a certain number of loci, this would, in our opinion, still provide us with a reliable estimate of population statistics for the whole set.

3.2. Linkage analysis

For performing the two-point linkage analysis (SI S6), we used only 123 polymorphic loci excluding all monomorphic markers at the same time. The loci for which we obtained small numbers of genotypes should be discarded, as well. If we remove the data for loci genotyped, say, in less than 20 individuals, we will have 118 markers identified so far, which could be informative for the linkage analysis.

As a next planned step, we will complete the genetic linkage analysis with one of the available software packages, identify probable linkage groups, verify the marker order and play around with the LOD value. Also, we will look into a probability of linking the chondrodystrophy trait to any linkage group.
