# Supplementary material for: Towards a Genetic Linkage Map of the California Condor, an Endangered New World Vulture Species
Source: Animals (Basel). 2022 Nov 24;12(23):3266. doi: 10.3390/ani12233266 (PMC9739316; doi:10.3390/ani12233266)
Supplement: Supplementary file 1 [file animals-12-03266-s001.zip › Supplementary Information S6 revised.pdf]

Supplementary Information S6: Genetic linkage analysis in California condors

Linkage group 1

Linkage group 1 includes 1A, 1B and 1C. 1A has strong evidence of marker order, while 1B does not have strong ordering evidence and 1C has no ordering information within this group. 1A and 1B could not be ordered relative to each other, and 1c has conflicting evidence about linkage to 1A or 1B.

115Aa 120A 153F 195F 38F 63G 66A 64G 9Fb 188F 129H 17G

Linkage group 1A

Ordering reliability: likelihood ratio (best order)/(2nd best order) = 239:1.

Sex_averaged map (recomb. frac., Kosambi cM):

7	115Aa			0.0
		0.03	3.3	
43	38F			3.3
		0.07	6.7	
31	195F			10.0

* denotes recomb. frac. held fixed in this analysis

log10_like = 11.66

Sex-specific map (recomb. frac., Kosambi cM -- female, male):

7	115Aa			0.0			0.0
		0.06	5.9		0.00	0.2	
43	38F			5.9			0.2
		0.04	3.5		0.10	10.1	
31	195F			9.4			10.3

* denotes recomb. frac. held fixed in this analysis

log10_like = 11.94

number of loci to flip = 2

Original order, & its log10_likelihood, followed by

flipped orders, with their relative log10_likelihoods

(= log10_like[orig] - log10_like[curr])

```
  7 43 31     11.66

 43  7  -      2.38
  - 31 43      3.56
```

Linkage group 1B

Sex_averaged map (recomb. frac., Kosambi cM):

```
  8   120A                      0.0
                   0.01    1.3
115   66A                       1.3
                   0.02    2.4
 52   63G                       3.7
                   0.00    0.0
 21   153F                      3.7
                   0.06    5.8
  1   101H                      9.5
```

		0.08	7.9		
68	9Fb			17.4	
		0.04	3.8		
27	17G			21.2	

* denotes recomb. frac. held fixed in this analysis

log10_like = -5.34

Sex-specific map (recomb. frac., Kosambi cM -- female, male):

8	120A			0.0			0.0
		0.00	0.0		0.03	2.9	
115	66A			0.0			2.9
		0.01	1.4		0.04	3.5	
52	63G			1.4			6.4
		0.00	0.0		0.00	0.0	
21	153F			1.4			6.4
		0.02	2.2		0.03	2.6	
1	101H			3.6			9.0
		0.00	0.0		0.21	22.9	
68	9Fb			3.6			31.9
		0.04	3.9		0.03	3.4	
27	17G			7.5			35.4

* denotes recomb. frac. held fixed in this analysis

log10_like = -3.76

115Aa

```
    8   115  52  21  1   68  27
  X   X   X       X   X
```

-11.30

-14.11

-12.99

-12.36

-12.89

38F

```
    8   115  52  21  1   68  27
  X   X   X   X   X
```

-7.74

-6.50

-6.50

-7.89

-7.90

```
195F
    8   115  52  21  1   68  27
  X   X           X   X

-12.42
-13.20
-14.78
-15.24
number of loci to flip = 5


Original order, & its log10_likelihood,  followed by
flipped orders, with their relative log10_likelihoods
(= log10_like[orig] - log10_like[curr])

  8115 52 21  1 68 27     -5.34

  1 21  -115  8  -  -      1.04
  1 21  -  8115  -  -      1.76
  1 21115 52  8  -  -      2.16
  1 21  8 52115  -  -      2.87
  1 21115  8 52  -  -      2.85
  1 52 21115  8  -  -      1.04
  1 52 21  8115  -  -      1.76
  1  - 21 52  8  -  -      2.17
  1  8 21 52115  -  -      2.04
  1  - 21  8 52  -  -      2.85
```

1	8	21	115	52	-	-	2.74
1	52	115	-	8	-	-	1.04
1	52	8	-	115	-	-	1.76
1	-	-	-	8	-	-	2.16
1	8	-	-	115	-	-	2.05
1	-	8	-	52	-	-	2.85
1	8	115	-	52	-	-	2.75
1	52	115	8	21	-	-	2.57
1	52	8	115	21	-	-	1.76
1	8	-	115	21	-	-	2.04
1	-	8	52	21	-	-	2.85
1	8	115	52	21	-	-	2.74
52	1	21	115	8	-	-	2.40
115	1	21	52	8	-	-	2.40
-	1	21	52	115	-	-	2.40
52	1	115	-	8	-	-	2.40
115	1	-	-	8	-	-	2.40
-	1	-	-	115	-	-	2.41
-	1	-	115	21	-	-	2.40
21	52	1	115	8	-	-	2.40
21	-	1	52	8	-	-	2.40
52	21	1	115	8	-	-	2.40
115	21	1	52	8	-	-	2.40
-	52	1	-	115	-	-	2.16
-	-	1	-	52	-	-	2.16
-	52	1	115	21	-	-	2.16
-	-	1	52	21	-	-	2.16
21	-	-	1	8	-	-	2.56

115	21	-	1	8	-	-	2.56
-	21	-	1	115	-	-	2.16
-	21	115	1	52	-	-	2.16
115	52	21	1	8	-	-	2.56
-	52	21	1	115	-	-	2.16
-	-	21	1	52	-	-	2.16
-	-	-	1	21	-	-	2.76
21	52	115	8	-	-	-	1.21
21	52	8	115	-	-	-	1.61
21	-	-	8	-	-	-	0.77
21	8	-	115	-	-	-	2.54
21	-	8	52	-	-	-	0.98
21	8	115	52	-	-	-	1.92
52	21	115	8	-	-	-	1.22
52	21	8	115	-	-	-	1.61
115	21	-	8	-	-	-	0.78
-	21	-	115	-	-	-	0.62
115	21	8	52	-	-	-	0.98
-	21	115	52	-	-	-	-0.00
52	-	21	8	-	-	-	1.21
52	8	21	115	-	-	-	1.61
115	52	21	8	-	-	-	0.77
-	52	21	115	-	-	-	0.63
115	8	21	52	-	-	-	0.98
-	-	21	52	-	-	-	0.00
52	-	8	-	-	-	-	2.28
52	8	115	-	-	-	-	1.61
115	52	8	-	-	-	-	1.76

-	52	115	-	-	-	-	0.62
115	8	-	-	-	-	-	0.98
-	21	-	115	68	1	-	2.79
-	21	115	52	68	1	-	2.67
-	52	21	115	68	1	-	2.79
-	-	21	52	68	1	-	2.68
-	52	115	-	68	1	-	2.79
-	-	-	-	68	1	-	2.67
-	-	-	27	21	1	68	2.47
-	-	21	52	27	-	1	2.71
-	-	-	-	27	-	1	2.71
-	-	21	52	27	1	68	1.25
-	-	-	-	27	1	68	1.24
-	-	21	52	68	27	1	2.31
-	-	-	-	68	27	1	2.30
-	-	1	-	52	27	68	2.54
-	-	1	52	21	27	68	2.54
-	-	21	1	52	27	68	2.54
-	-	21	52	-	27	68	0.56
-	-	-	-	-	27	68	0.56

Linkage group 1C

129H 64G 188F

These three markers could be the same marker, but had conflicting evidence about linkage to group 1A or 1B.

Linkage group 2

Ordering reliability: likelihood ratio (best order)/(2nd best order) = 707:1.

```
39F   109D  41A

Sex_averaged map (recomb. frac., Kosambi cM):


 44   39F                          0.0

                    0.16   16.7

  4   109D                        16.7

                    0.09    8.6

109   41A                         25.3


* denotes recomb. frac. held fixed in this analysis



log10_like = -28.71


Sex-specific map (recomb. frac., Kosambi cM -- female, male ):

 44   39F                          0.0                            0.0

                    0.12   12.2                   0.20   21.7

  4   109D                        12.2                           21.7

                    0.06    6.5                   0.11   11.2

109   41A                         18.7                           32.9


* denotes recomb. frac. held fixed in this analysis


log10_like = -28.27

135A

    44  4   109

          X   X
```

-38.05

-37.03

44   39F

  4   109D

109   41A

number of loci to flip = 2

Original order, & its log10_likelihood, followed by

flipped orders, with their relative log10_likelihoods

(= log10_like[orig] - log10_like[curr])

 44 4 109   -28.71

  4 44 -   8.00

  -109 4   2.85

Linkage group 3

21F   79H D10   D6

no ordering info

Linkage group 4

47G 92D   176A

no ordering info

Linkage group 5

157F 145A 167A

no ordering info

Linkage group 6

124D 157G 125G

no ordering info

Linkage group 7

20D 58F 74F

no ordering info

Linkage group 8

B7 H238

no ordering info

Linkage group 9

143H 111A

no ordering info

Linkage group 10

121A 33A

no ordering info

Linkage group 11

D24 58A

no ordering info

Linkage group 12

Ch262-87L14_2 D126

no ordering info

Linkage group 13

112G D9

no ordering info
